# Toughening Polyamidoamine Hydrogels through Covalent Grafting of Short Silk Fibers

**DOI:** 10.3390/molecules27227808

**Published:** 2022-11-12

**Authors:** Filippo Maggi, Amedea Manfredi, Federico Carosio, Lorenza Maddalena, Jenny Alongi, Paolo Ferruti, Elisabetta Ranucci

**Affiliations:** 1 Dipartimento di Chimica, Università degli Studi di Milano, Via C. Golgi 19, 20133 Milano, Italy; 2 Dipartimento di Scienza Applicata e Tecnologia, Politecnico di Torino, Alessandria Campus, Viale Teresa Michel 5, 15121 Alessandria, Italy

**Keywords:** silk, polyamidoamine, composite hydrogels, reinforced hydrogels

## Abstract

Linear amphoteric polyamidoamines (PAAs) are usually water-soluble, biodegradable and biocompatible. Crosslinked PAAs form in water hydrogels, retaining most of the favorable properties of their linear counterparts. The hydrogels prepared by the radical post-polymerization of the oligo-α,ω-bisacrylamido-terminated PAA called AGMA1, obtained by the polyaddition of 4-aminobutylguanidine (agmatine) with 2,2-bis(acrylamido)acetic acid, exhibit excellent cell-adhesion properties both in vitro and in vivo. However, due to their low mechanical strength, AGMA1 hydrogels cannot be sewn to biological tissues and need to be reinforced with fibrous materials. In this work, short silk fibers gave excellent results in this sense, proving capable of establishing covalent bonds with the PAA matrix, thanks to their lysine content, which provided amino groups capable of reacting with the terminal acrylamide groups of the AGMA1 precursor in the final crosslinking phase. Morphological analyses demonstrated that the AGMA1 matrix was intimately interconnected and adherent to the silk fibers, with neither visible holes nor empty volumes. The silk/H-AGMA1 composites were still reversibly swellable in water. In the swollen state, they could be sewn and showed no detachment between fibers and matrix and exhibited significantly improved mechanical properties compared with the plain hydrogels, particularly as regards their Young’s modulus and elongation at break.

## 1. Introduction

Hydrophilic polymers, which in linear form are water-soluble, if crosslinked, give rise to insoluble materials, which in aqueous media absorb large quantities of water while retaining their three-dimensional structure; these are usually referred to as hydrogels [1,2]. Thanks to their high water-content, hydrogels are soft viscoelastic materials endowed with physical properties, including nutrient permeability and low interfacial tension, resembling those of soft biological tissues. If the related linear polymers are biocompatible and biodegradable, hydrogels can be considered for use as scaffolds for soft tissue engineering. Indeed, many different hydrogels, both natural and synthetic, have been extensively studied for this purpose [3,4,5].

Linear polyamidoamines (PAAs) are a versatile family of synthetic polymers prepared by the aza-Michael polyaddition of amines with bisacrylamides [6]. Crosslinked PAAs can be obtained by different methods, for instance by using multifunctional amines as co-monomers or by preparing PAA pre-polymers with terminal acrylamide double bonds and then triggering their radical polymerization by conventional techniques, such as UV irradiation or the addition of radical initiators. It may be observed that, in this process, the acrylamide terminals of the oligomeric PAA precursors are still susceptible to undergoing aza-Michal addition reaction with amines. Amphoteric PAAs, containing both amino and carboxyl groups in their repeat units, have proven in several instances to be highly biocompatible [7]. As a rule, PAAs are highly hydrophilic and, if linear, are in most cases water-soluble, whereas if crosslinked, they give hydrogels [8,9].

An amphoteric but prevailingly cationic hydrogel based on a PAA named AGMA1, carrying in its repeat units both carboxyl and guanidine groups, combined bioactivity with biocompatibility and biodegradability [10,11,12]. AGMA1 hydrogels, as their linear counterpart [13], have excellent cell adhesion properties [14,15,16,17]. When tested in vivo in a rat model, as conduits for sciatic nerve regeneration, they have been remarkably successful [18]. An implanted AGMA1 hydrogel tube was indeed completely resorbed after 90 days implantation. The nerve function was fully restored, with no signs of inflammation or neuroma, and the rat fully recovered the function of its hind leg. However, the AGMA1 hydrogel tubes could only be fixed to the surrounding tissues with fibrin glue, because they were too soft to sew. Montmorillonite-reinforced AGMA1 hydrogels exhibited shear storage modulus twenty times higher than that of the virgin hydrogels, but even they were not yet suturable [19].

It is possible to prepare fiber-reinforced composite hydrogels that combine the best properties of both components and meet the strength and toughness requirements indispensable for various technological applications [20,21], including as scaffolds for tissue engineering [22,23,24,25,26]. For instance, it was found that AGMA1 hydrogels, which had superior mechanical properties and were suturable, could be obtained by reinforcing them with an electrospun poly-L-lactic acid (PLLA) mat, modified on the surface with amino groups by means of nitrogen plasma [27] to allow the covalent grafting of AGMA1 on the PLLA surface. It is of note that untreated PLLA mats detached from the PAA matrix upon swelling in water.

Silk fibroin, a natural protein extracted from *Bombyx mori* cocoons, is endowed with outstanding mechanical properties, biocompatibility, and bioresorbability and has attracted significant attention as a biomedical material [28,29,30,31], including as a scaffold for tissue engineering [32,33,34,35]. The lysine residues present in silk fibroin provide free amino groups capable of participating in the aza-Michael polyaddition process leading to PAAs. Therefore, it was hypothesized that silk fibers could act as effective reinforcing agents for PAA hydrogels by being able, without any prior treatment, to establish covalent bonds with them during their preparation. This assumption turned out to be correct. A mat of short-cut silk fibers was impregnated with a water solution of an AGMA1 oligomer carrying α,ω-acrylamide-terminated groups and subsequently subjected to UV irradiation. The result was a silk-reinforced hydrogel reversibly swellable in water (similarly to the plain hydrogel), which remained apparently unaffected by unlimited numbers of water-swelling and de-swelling cycles. In the swollen state, the silk/AGMA1 composite hydrogels showed much improved mechanical properties and were sufficiently strong to allow sewing. The aim of the research was to establish a general procedure for toughening AGMA1 hydrogels and making them suturable.

## 2. Results

### 2.1. Rationale

The approach devised to toughen AGMA1 hydrogels and make them resistant to stitching was to embed a silk mat obtained from short silk fibers (0.3–2.0 mm) in the AGMA1 hydrogel, in the meantime allowing the establishment of covalent bonds between them. The hydrogel matrix was obtained by UV-induced radical polymerization of α,ω-acrylamide-terminated AGMA1 oligomeric precursors. The silk/AGMA1 covalent bonds were established during the crosslinking process, by the aza-Michael reaction of the amine residues present in the L-lysine units with a portion of the terminal acrylamide groups. The final silk/AGMA1 composite hydrogels potentially combined the biocompatibility, biodegradability, and cellular adhesiveness of both components. In fact, they were designed to be used as scaffolds for tissue engineering, thanks to the possibility that they could mimic the combination of the mechanical properties of the gel and fibrous component of the extracellular matrix.

### 2.2. Synthesis of Plain AGMA1 Hydrogels (H-AGMA1)

Plain AGMA1 hydrogels (H-AGMA1) were prepared in order to compare their physical properties with those of the silk/reinforced AGMA1 hydrogels (silk/H-AGMA1).

**Scheme 1:**
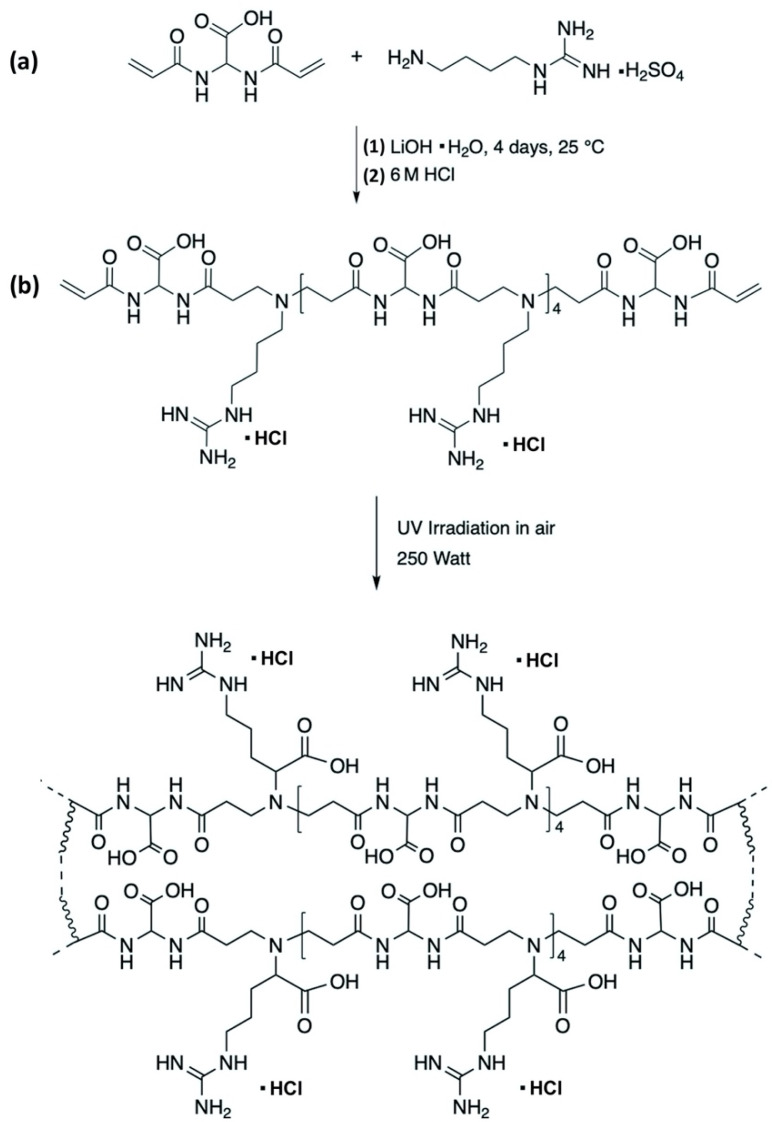
Preparation of the H-AGMA1 hydrogel: synthesis of the AGMA1 oligomer (**a**); cross-linking reaction (**b**).

**Figure 1:**
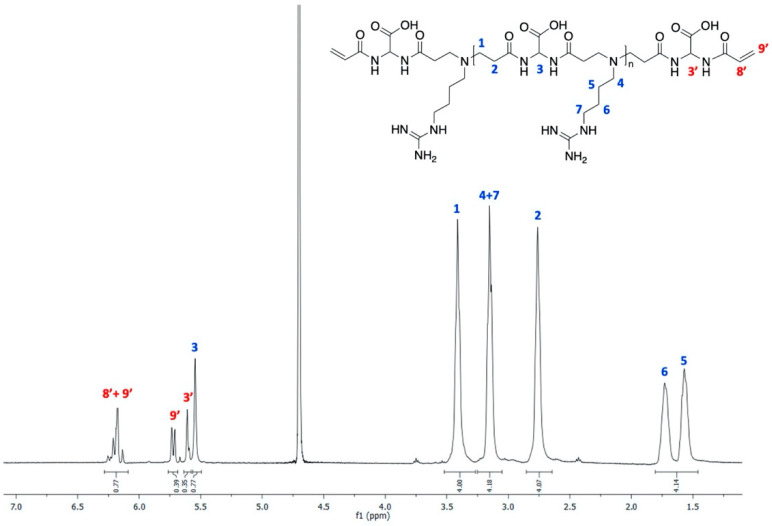
^1^H-NMR spectrum of the α,ω-bisacrylamide-terminated AGMA1 oligomer run in D_2_O using a 400.13 MHz Brüker Avance Spectrometer. The blue colored numbers refer to the protons of the internal repeat units; the red colored numbers refer to the protons of the terminals.

**Figure 2:**
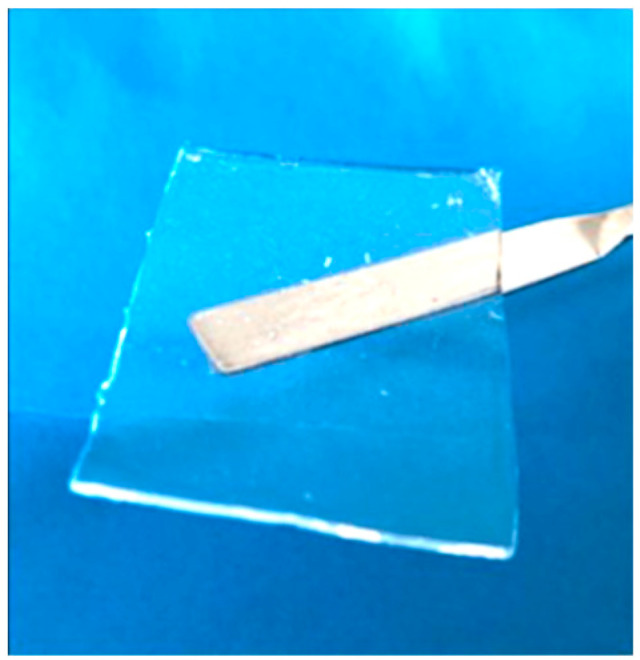
Freshly isolated H-AGMA1 hydrogel sample.

H-AGMA1 preparation consisted of two main steps. The first was the synthesis of an α,ω-acrylamide-terminated AGMA1 oligomeric precursor amenable to be crosslinked by radical polymerization. This AGMA1 precursor was prepared by introducing in the polymerization mixture 20% excess, on a molar basis, 2,2-bis(acrylamido)acetic acid (BAC), with respect to 4-aminobutylguanidine (agmatine) (Scheme 1a). In fact, in bifunctional stepwise polymerization, the products of stoichiometrically unbalanced polymerization experiments are oligomers α,ω-terminated with the excess function [36]. The AGMA1 reactive oligomers were obtained as 52% (*w*/*w*) aqueous solutions. The structure of the AGMA1 precursor was confirmed by means of ^1^H-NMR spectroscopy (Figure 1). The second preparation step consisted of triggering the radical polymerization of the AGMA1 reactive oligomer, via the terminal acrylamide groups, by UV irradiation (Scheme 1b). The crosslinking reaction was carried out inside a glass mold, giving a soft 1 mm thick hydrogel sheet (Figure 2).

### 2.3. Synthesis of Silk/AGMA1 Composite Hydrogels (Silk/H-AGMA1)

**Figure 3:**
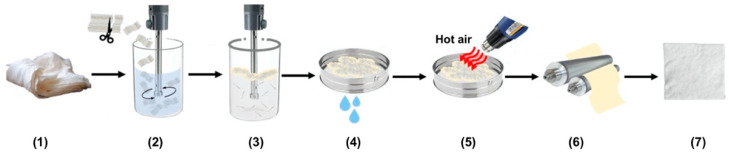
Scheme of the preparation of a short-fiber silk mat: (1) raw degummed silk; (2) dispersing degummed silk chops in water; (3) grinding silk fibers into short-fibers (length 0.3–2 mm); (4) filtering the obtained silk mash through a 250 μm mesh sieve; (5) drying with hot air; (6) compressing the silk mat through counter-rotating rolls; (7) final mat.

**Figure 4:**
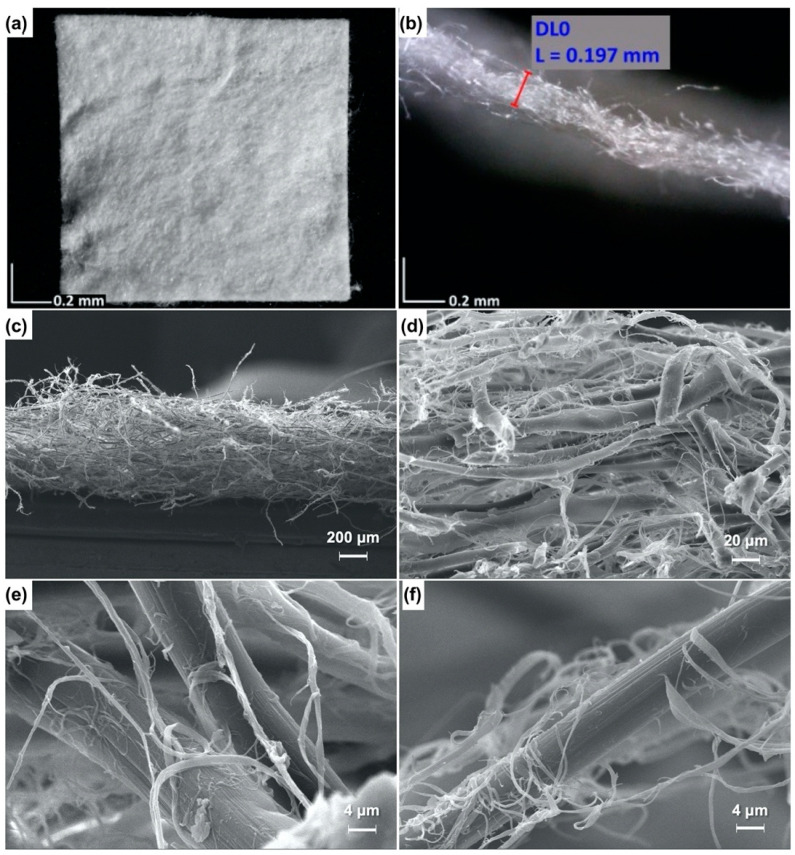
Optical microscope (**a**,**b**) and SEM micrographs of the silk mat at different magnifications ((**c**): 100×; (**d**): 1000×; (**e**): 5000×; (**f**): 5000×). The continuous fibers represent fibroin, whereas the filaments are sericin residues.

The silk/H-AGMA1 preparation consisted of three main steps. In the first step, an approximately 200 μm thick silk mat was obtained by first preparing a fine aqueous dispersion of short silk fibers (0.3–2.0 mm in length and 5–15 µm in diameter) by grinding degummed raw silk, then filtering the resultant short fibers through a 250 μm stainless steel mesh, drying, and finally pressing through two counter-rotating rolls (Figure 3). The resulting mat looked like a “felt” (Figure 4a,b), whose morphology, analyzed by Scanning Electron Microscopy (SEM) (Figure 4c–f), consisted of a dense interweaving of fibroin fibers with some residual serine filaments. The porous structure of the silk mat favored integration between the silk fibers and the AGMA1 hydrogel matrix.

**Figure 5:**
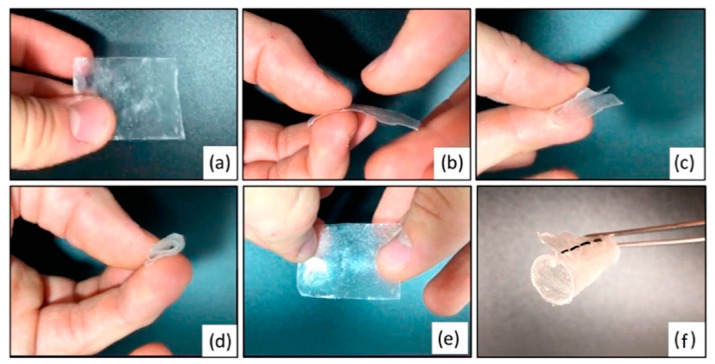
Silk/H-AGMA1 composite hydrogel: just retrieved from the mold (**a**); undergoing folding (**b**–**d**); extended after folding (**e**); sewn (**f**).

In a second step, the silk mat was impregnated with the same α,ω-bisacrylamide-terminated AGMA1 oligomer solution used in the preparation of H-AGMA1 (see Section 3.2) in a mold consisting of two silanized glass plates with a 130 μm plastic frame (see Section 3.4) and finally crosslinked by UV irradiation. The composition, on a weight basis, of the obtained composite hydrogel was 30% silk, 20% AGMA1, and 50% water. By gently detaching the samples from the glass plates and extensively washing with ultrapure water, cloudy, flexible, pliable, and macroscopically homogeneous silk/H-AGMA1 composite hydrogels were obtained, which could withstand extensive handling, including sewing, without any visible damage (Figure 5).

### 2.4. Morphological Characterization of Silk/H-AGMA1 Composite Hydrogels

**Figure 6:**
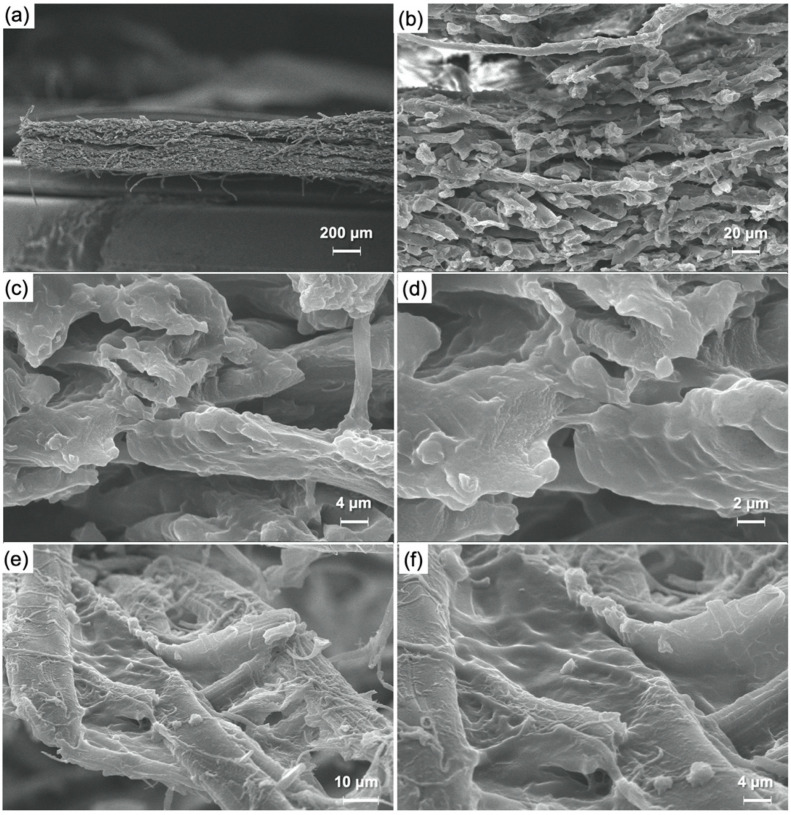
SEM micrographs of silk/H-AGMA1 composite hydrogels at different magnifications: (**a**): 100×; (**b**): 1000×; (**c**): 5000×; (**d**): 10000×; (**e**): 2500×; (**f**): 5000×.

The morphological characterization of silk/H-AGMA1 samples was investigated by means of SEM (Figure 6). In the composite, it is apparent that the silk fibers are completely and homogeneously embedded in the H-AGMA1 matrix. Indeed, both low-magnification and high-magnification pictures show evidence of a highly interconnected and integrated system where the fibers and the hydrogel matrix are perfectly adherent to each other, with no visible holes or gaps between adjacent fibers.

### 2.5. Water Uptake of H-AGMA1 and Silk/H-AGMA1 Hydrogels

**Figure 7:**
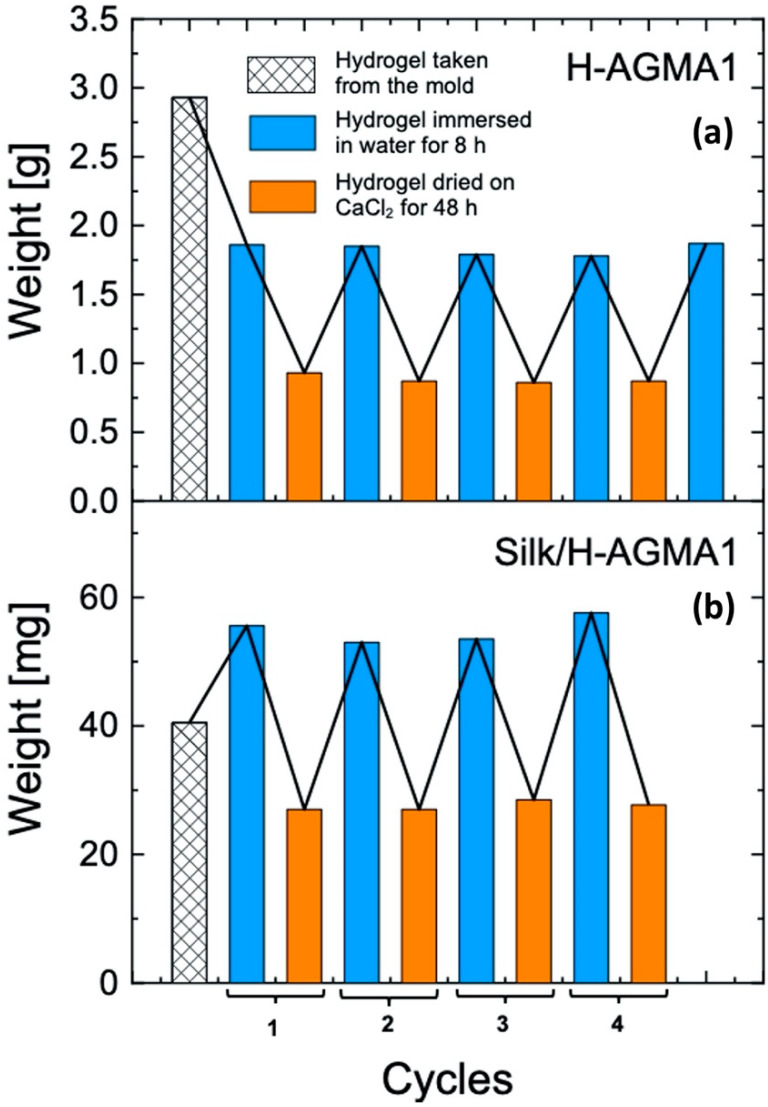
Water uptake of H-AGMA1 (**a**) and silk/H-AGMA1 (**b**) hydrogels.

The results of the water uptake tests carried out on H-AGMA1 and silk/H-AGMA1 hydrogels are shown in Figure 7. It may be noticed that in the first water absorbing cycle, the H-AGMA1 sample (Figure 7a) underwent a significant weight loss (approximately 40%), probably due to the extraction of soluble fractions. Nonetheless, it showed complete reversibility in the subsequent multiple swelling–drying cycles, with excellent resistance to the osmotic stress encountered in the process. In the case of the silk/H-AGMA1 composite hydrogel, weight loss was never observed after the first water absorbing cycle. This behavior was ascribed to the efficient covalent grafting of the matrix onto the reinforcing agent. In addition, the swelling behavior of the silk/H-AGMA1 samples was not affected by the presence of the reinforcing material, as it exhibited a perfect reversibility of the swelling/deswelling capacity after multiple cycles, as observed for the plain H-AGMA1 hydrogel. Furthermore, it is worth mentioning that the silk/AGMA1 samples could stand several weeks immersed in water without any evidence of detachment of the AGMA1 matrix from the silk fibers, further confirming the existence of covalent bonds between them.

### 2.6. Thermal Stability of the Silk/H-AGMA1 Composite Hydrogels

**Figure 8:**
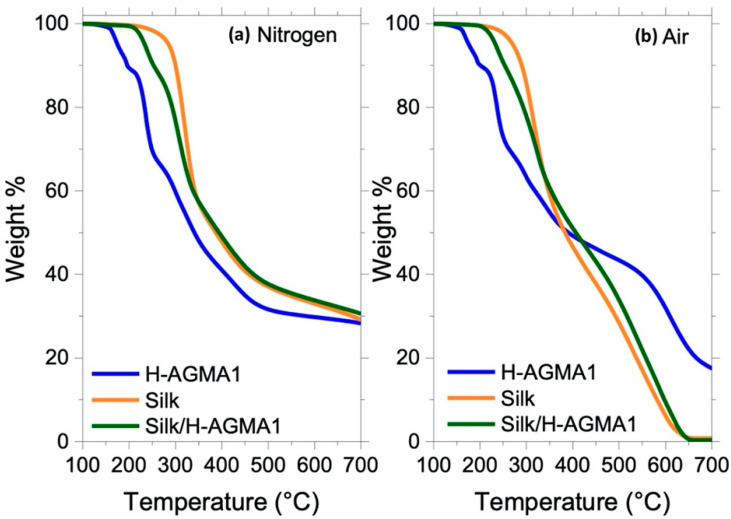
TG thermograms of H-AGMA1, silk mat, and silk/H-AGMA1 in nitrogen (**a**) and air (**b**). All hydrogel samples were dried in isothermal conditions at 100 °C for 3 min prior to the heating run.

The thermal stability of the silk/H-AGMA1 composite hydrogels was investigated by means of Thermogravimetric Analysis (TGA) and compared with those of both composite constituents, namely the H-AGMA1 matrix and the silk mat. The TG thermograms and the collected results are reported in Figure 8 and Table 1, respectively.

In nitrogen, H-AGMA1 decomposed through a multimodal mechanism that took place in four steps, two of which were in the 160–250 °C range, and the remaining two, representing maximum weight losses, were centered at 235 °C and 304 °C (Figure 8a). Noticeably, the decomposition onset temperature at 10% weight loss, T_onset10%_, was 197 °C, that is, remarkably lower than in the reinforced hydrogel. As regards the silk mat, the T_onset10%_ was much higher than that of H-AGMA1 (302 vs. 197 °C, Table 1), and its decomposition occurred in two steps according to the literature [37,38], leaving a residual mass fraction at 700 °C comparable to that of H-AGMA1 (29 vs. 28%). Noticeably, the thermogram of the silk/H-AGMA1 composite was placed in an intermediate position with respect to those of both constituent, silk and H-AGMA1, in the 100–320 °C range, while it almost completely overlapped that of the silk mat above 320 °C. Moreover, its residual mass fraction was slightly higher than those of both H-AGMA1 and the silk mat (Table 1). The observed similarities between the thermal profile of silk/H-AGMA1 and that of the silk mat can only be explained by extremely strong interactions between the two components of the composite hydrogel, further supporting the hypothesis of the occurrence of covalent bonds between them.

**Figure 9:**
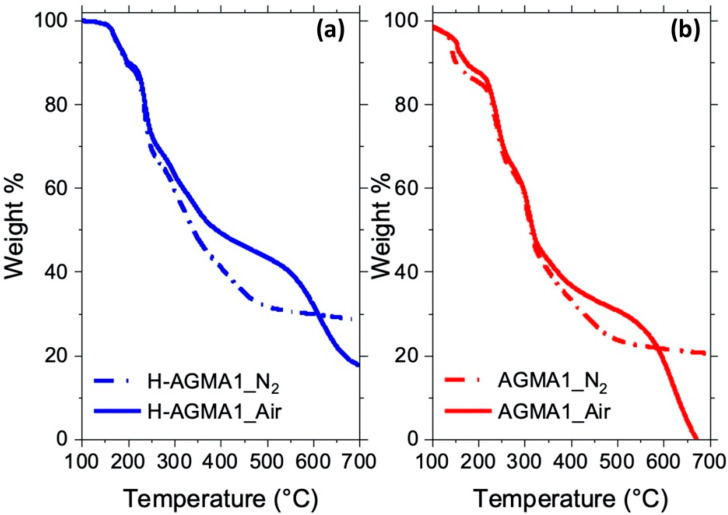
Comparison between the TG curves of H-AGMA1 hydrogel (**a**) and linear AGMA1 (**b**) in nitrogen and air.

In air, the thermogram of the H-AGMA1 hydrogel overlapped that in nitrogen in the 100–250 °C range (Figure 9). At higher temperatures the two curves diverged, and the hydrogel turned out to be more stable in air than in nitrogen, as also evidenced in Figure 9, where the TG curves of the H-AGMA1 hydrogel (a) and the linear AGMA1 (b) in nitrogen and air are reported, in line with the previously observed behavior of linear AGMA1. In between 300 and 600 °C, the H-AGMA1 hydrogel underwent oxidation, producing a thermally stable carbonaceous char thanks to the phenomenon of intumescence [39], which is an intrinsic characteristic of linear polyamidoamines (PAAs) [40]. This structure inhibits the transfer of mass, volatile combustible substances and oxygen from the atmosphere to the polymer, thus inhibiting further oxidation. This phenomenon does not occur in nitrogen, as observable in Figure 9.

Comparing the thermograms of H-AGMA1, silk mat, and silk/H-AGMA1 in air, it is possible to observe a similar behavior to that already described in nitrogen, except for the oxidation step occurring between 400 and 650 °C (Figure 8b). In the case of H-AGMA1, this step proceeded through an intumescence step, providing a high residual mass fraction of about 18% at 700 °C. The thermogram of the silk/H-AGMA1 composite was instead placed in an intermediate position with respect to those of both the constituents, silk and H-AGMA1, in the range 100–320 °C, as already observed in nitrogen, while it almost completely overlapped with that of the silk mat above 320 °C. In both samples, the further oxidation of the residue formed at 320 °C led to a negligible final residual mass at 700 °C. Finally, the T_onset10%_ and T_max_ values of all of them in air were significantly comparable with those in nitrogen, as well (Table 1).

### 2.7. Tensile Properties of Silk/H-AGMA1 Hydrogels in the Swollen State

**Figure 10:**
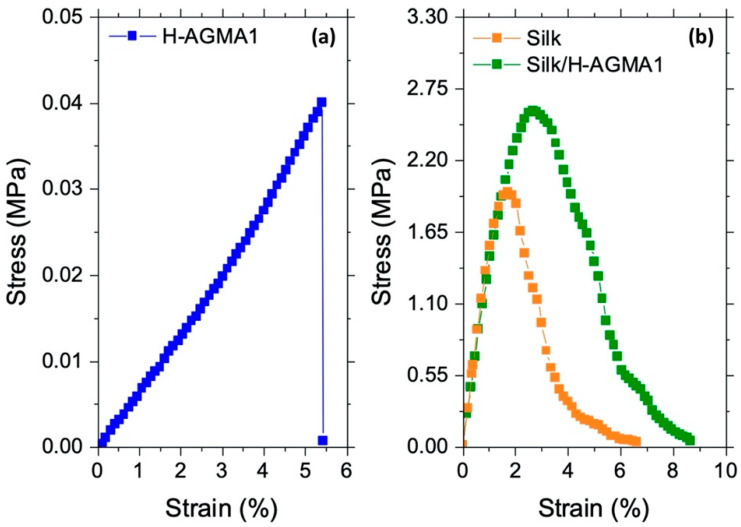
Stress–strain curves of H-AGMA1 hydrogel (**a**) and silk mat and silk/H-AGMA1 hydrogel (**b**). Both samples were analyzed swollen in water.

The tensile properties of water-swollen silk/H-AGMA1 were analyzed and compared with those of both composite constituents, that is, the H-AGMA1 hydrogel matrix and the silk mat, both in the fully hydrated state. It should be noted that the virgin and composite hydrogel samples used in the mechanical tests had a fairly different thickness (about 550 μm versus 130 μm), since at lower thicknesses the virgin hydrogels were too soft to resist handling. The resulting stress–strain curves are shown in Figure 10. It was evident that the H-AGMA1 hydrogel was a rigid material with a low value of elastic modulus (0.3 MPa, Table 2). It withstood an ultimate tensile strength of 0.10 N and a maximum stress of 0.04 MPa, after which it broke following a maximum deformation of 5.3%. Conversely, the silk/H-AGMA1 hydrogel was a tough material, whose stress–strain curve had a similar trend to that of the silk mat. Its elastic modulus (163 MPa, Table 2) and ultimate tensile strength (1.95 MPa, Table 2) were indeed significantly higher than those of plain H-AGMA1 and not significantly lower than those of silk (192.7 MPa and 1.95 N, respectively Table 2). In addition, the maximum stress and elongation at break were not only remarkably higher than those of H-AGMA1 but also appreciably higher than those of silk (2.58 MPa vs. 1.96 MPa and 8.2% vs. 6.8%, respectively).

**Figure 11:**
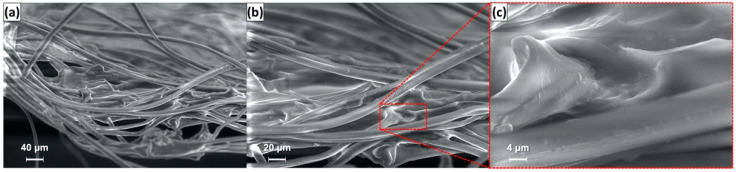
SEM micrographs at different magnifications of the fracture surface of the silk/H-AGMA1 specimen after mechanical tests: (**a**): 500×; (**b**): 1000×; (**c**): 5000×.

The morphological analysis of the fracture surface of the silk/H-AGMA1 composite hydrogel, carried out by SEM (Figure 11), revealed that the silk fibers still appeared perfectly covered by and adherent to the H-AGMA1 hydrogel matrix. This result confirms the hypothesis of the establishment of covalent bonds between the silk fibers and the hydrogel matrix and their key role in the reinforcing action in the composite.

## 3. Materials and Methods

Lithium hydroxide monohydrate (≥98%) (LiOH·H_2_O) was purchased from Fluka (Milano, Italy); 4-aminobutylguanidine (agmatine) sulphate (95%) was purchased from Enamine (Riga, Latvia); 2,2-bis(acrylamido)acetic acid (BAC) (>98%) was synthesized as previously described [7]. Ultrapure water for HPLC was purchased from CHROMASOLV™ (Honeywell, Charlotte, NC, USA); deuterated water (D_2_O) (99.9% D), fuming hydrochloric acid (≥37% in water), and acetone (99.5%) purchased from Sigma Aldrich (Milano, Italy); absolute ethanol (99.5%) was purchased from Scharlab S.L. (Barcelona, Spain); physiological pH 7.4 buffer solution (disodium hydrogen phosphate/potassium dihydrogen phosphate) was purchased from Merck (Milano, Italy); pure degummed raw silk was purchased from Beesybee Fibers (Bolinas, CA, USA). A stainless-steel wire mesh sieve (mesh 60, 250 μm) was purchased from Giuliani Tecnologie S.r.l. (Torino, Italy).

The ^1^H-NMR spectra were obtained using a Brüker Avance DPX-400 NMR spectrometer (Milano, Italy) operating at 400.13 MHz. The number of scans (32), relaxation delay (d1, 10.0 s), and receiver gain were automatically measured and set by the instrument. Analyses were conducted in D_2_O, adjusting the pH to 4.0 with D_2_O solutions of DCl.

### 3.1. Preparation of the Silk Mat

Degummed raw silk (1.0 g) was cut into 10 mm × 5 mm chops and then suspended in ultrapure water (400 mL) and ground for 5 min at 25 °C with an Ultra-Turrax^®^ IKA T25 disperser (IKA, Staufen, Germany) operating at 22–23 × 10^3^ rpm (Figure 3). A floating slurry of silk fibers of 0.3–2.0 mm length and 5–15 µm diameter was obtained. This slurry was then filtered under vacuum through a stainless-steel wire mesh with pore size 250 μm sieve, giving a silk mat that was first gently compressed with a spatula, then dried in a stream of hot air (100 °C × 5 min per side) to give a disc of 10 cm diameter that was further compressed through two counter-rotating rollers. The final disc (0.6 g, 60% yield) had an average final thickness of 236 ± 40 μm.

### 3.2. Synthesis of the α,ω-Bisacrylamide-Terminated AGMA1 Oligomer

2,2-Bis(acrylamido)acetic acid (BAC) (10.00 g, 49.30 mmol) and lithium hydroxide monohydrate (2.06 g, 49.30 mmol) were dissolved in water (20.00 g). Agmatine sulphate (9.48 g, 41.52 mmol) and lithium hydroxide monohydrate (1.74 g, 41.52 mmol) were then added. The reaction mixture was heated at 50 °C under stirring until complete dissolution, then maintained at 25 °C for 4 days in the dark. An aliquot (1 mL) of the solution was finally acidified to pH 4.5 with 6 M HCl and freeze-dried, and the solid obtained was analyzed by ^1^H-NMR (Figure 1). The remaining portion was used in the hydrogel preparation without further treatment.

### 3.3. Preparation of H-AGMA1 Hydrogel Samples

The H-AGMA1 hydrogel samples were obtained as sheets using as a mold two silanized 2 mm thick glass plates separated by a 1 mm thick silicone frame, leaving a void volume of 60 mm × 60 mm × 1 mm where the α,ω-bisacrylamide-terminated AGMA1 oligomer solution could be introduced. Before use, the glass plates were soaked in aqua regia for 5 h, extensively washed with water, dried, and then silanized by exposing to trichloromethylsilane vapors in a closed chamber for 3 days. They were subsequently soaked in toluene (20 mL), ethanol (2 × 20 mL), and then water (3 × 20 mL). The plates were finally gently wiped before use.

H-AGMA1 samples were obtained by injecting in the mold the desired amount (normally from 0.6 to 3 mL) of the α,ω-bisacrylamide-terminated AGMA1 oligomer solution (see Section 3.2) and then exposing both sides of the glass mold to UV irradiation with a 250 W HG200 Ultra (Jelosil S.r.l., Vimodrone, Milan, Italy) UV lamp (emission range 315–400 nm) placed at 15 cm distance for 30 min per side. The thickness of the hydrogel samples so obtained averaged 576 ± 74 μm, as determined using a Dino-Lite Edge digital microscope (AM7115MZT model) with 5-megapixel resolution (VWR International S.r.l, Milano, Italy).

### 3.4. Synthesis of Silk/AGMA1 Composite Hydrogels (Silk/H-AGMA1)

Silk-reinforced H-AGMA1 samples were obtained as 130 μm thick sheets using a mold similar to that used for the preparation of H-AGMA1 hydrogels (Section 3.3), but with a 130 μm thick polyethylene frame leaving a void volume of 50 mm × 30 mm × 0.13 mm.

The silk/AGMA1 hydrogels were obtained by first placing an aliquot of the α,ω-bisacrylamide-terminated AGMA1 oligomer solution (0.15 mL) in the open mold within the frame. A 45 mm × 24 mm silk mat strip (85 mg) was then inserted in the mold and lightly compressed to facilitate impregnation with the previously introduced AGMA1 solution. An additional aliquot of the AGMA1 solution (0.15 mL) was then added, and the mold was closed with a second glass plate and blocked, taking care to let out the excess AGMA1 solution to ensure that the silk was completely impregnated. The crosslinking reaction was subsequently triggered by UV irradiation as described above for the plain H-AGMA1 hydrogel. The weight and the thickness of the silk/AGMA1 hydrogel sample thus obtained were 200 mg and 130 μm, using a Dino-Lite Edge digital microscope (AM7115MZT model) with 5-megapixel resolution (VWR International S.r.l, Milano, Italy).

### 3.5. Water Uptake Tests

Water uptake tests were performed on freshly prepared hydrogel samples with dimensions 50 mm × 45 mm × 1 mm (2.30 g) in the case of plain H-AGMA1 and 25 mm × 10 mm × 0.15 mm (40 mg) in the case of silk/composites H-AGMA1. These tests consisted of a series of water absorption/drying cycles, each performed by first soaking the hydrogel samples in ultrapure water for 8 h, gently blotting with paper towels, and then weighing. The swollen samples were then dried in a desiccator containing calcium chloride at 25 °C up to constant weight (approximately 48 h). The water uptake of the hydrogel samples (WU%) was calculated using Equation (1):

(1) $$\mathrm{WU}\%=\frac{W_{t}-W_{o}}{W_{o}}\times 100$$

where W_o_ = weight of the dry hydrogel and W_t_ = weight of the swollen sample.

### 3.6. Thermogravimetric Analysis

The thermal and thermo-oxidative stability of the silk mat and of the H-AGMA1 samples was evaluated by thermogravimetric (TG) analyses in nitrogen and in air, respectively, from 100 to 700 °C, with a heating rate of 10 °C min^−1^. A TAQ500 analyzer was used, placing the samples (ca. 10 mg) in open alumina pans, in an inert or oxidative atmosphere (gas flow: 60 mL min^−1^). In the following, T_onset10%_ and T_max_ will be defined as the temperature corresponding to a weight loss of 10% and the maximum weight loss rate, respectively. All hydrogel samples were dried in isothermal conditions at 100 °C for 3 min prior to the heating run. TG measurements were duplicated in order to assess their reproducibility.

### 3.7. Scanning Electron Microscopy

The surface morphology of the silk mat and of the H-AGMA1 samples, also after mechanical tests, was studied using a LEO-1450VP Scanning Electron Microscope (beam voltage: 20 kV). An X-ray probe (INCA Energy Oxford, Cu-Ka X-ray source, kα = 1.540562 Å) and a working distance of 8.40 mm were used to perform elemental analysis. Samples (5 mm × 5 mm) were cut and fixed to conductive adhesive tapes and gold-metallized.

### 3.8. Tensile Tests

Tensile tests were performed on AGMA1 hydrogels using an INSTRON 5966 series 3400 single column tensile testing machine equipped with a 50 N load cell and pneumatic side action clips. The sample thickness of all hydrogels and membranes was measured by using a digital micrometer (Juwel plus, Milano, Italy). The same speed program consisting of two different phases was used for all analyses. Initially, within the elastic range, we proceeded with a starting tensile speed of 1 mm min^−1^. Subsequently, upon reaching 0.3% strain, the speed was increased to 3 mm min^−1^. The hydrogels, with rectangular dimensions, were all tested under maximum swelling conditions. In contrast to the speed schedule, which is the same for all tests and sample types, the initial preload and specimen size were evaluated on the individual tests.

## 4. Conclusions

The results reported in this paper justify the following conclusions. Degummed raw silk has L-lysine-derived amine groups that are liable to undergo aza-Michael reaction with the acrylamide terminals of PAA oligomers purposely prepared by setting an unbalanced bis-acrylamide/amine molar ratio in the feed. In this work, this approach was applied to covalently bind short silk fibers to a hydrogel matrix through the UV-triggered radical polymerization of an α,ω-bisacrylamide-terminated amphoteric PAA called AGMA1 prepared by the polyaddition of 4-aminobutylguanidine with 2,2-bis(acrylamido)acetic acid. In detail, impregnating a silk mat obtained from a suspension of very short silk fibers with a concentrated aqueous solution of the oligomeric AGMA1 precursor, and subsequently exposing the resultant crude composite to UV irradiation, produced a tough silk-reinforced hydrogel. This composite hydrogel was still reversibly swellable in water, as was the corresponding unreinforced hydrogel, and remained unaffected after several water-uptake/drying cycles, showing no evidence of separation of its components after prolonged incubation in water. Moreover, the composite hydrogel showed much improved mechanical properties in the swollen state with respect to the virgin hydrogel, both in terms of Young’s modulus, ultimate strength, and elongation at break. Noticeably, it was strong enough to be suturable.

The hypothesis of covalent binding as a key factor to justify the remarkable dimensional and mechanical stability of silk/H-AGMA1 was also supported by the results of thermogravimetric and morphological analyses. The reinforced hydrogel was indeed significantly more stable in both nitrogen and air than the plain hydrogel, and its TG trace turned to be very close to that of silk. SEM analysis showed an intimate interconnection between fibers and the polymer matrix in the composite hydrogel, with no visible holes or void volumes, in line with the morphology observed after mechanical test. The appearance of the fracture surface of the composite showed no separation of the two constituent phases, confirming their remarkable integration.

All the above evidence leads to the conclusion that silk fibers are excellent reinforcing agents for PAA hydrogels and that they do not require any prior chemical treatment to form strong bonds with the PAA matrix and integrate with it.

## Figures and Tables

**Table 1:** Thermogravimetric data of H-AGMA1, silk mat, and silk/H-AGMA1 in nitrogen and air.

Sample	T_onset10%_^a^ [°C]	T_max1_^b^ [°C]	T_max2_^c^ [°C]	T_max3_^d^ [°C]	Residual Mass Fraction at 700 °C [%]
*Nitrogen*					
H-AGMA1	197	172/194	235	301	28
Silk	302	-	-	319/390	29
Silk/H-AGMA1 composite	251		239	307/414	31
*Air*					
H-AGMA1	201	165/194	236	295	18
Silk	290	-	-	320	0
Silk/H-AGMA1 composite	253	-	242	324	0

^a^ Onset decomposition temperature at 10% weight loss. ^b^ First temperature at maximum weight loss rate. ^c^ Second temperature at maximum weight loss rate. ^d^ Third temperature at maximum weight loss rate.

**Table 2:** Mechanical data of the silk/H-AGMA1 hydrogel compared with the composite constituents.

Sample	Length (mm)	Width (mm)	Thickness (mm)	Elastic Modulus (MPa)	Maximum Stress (MPa)	Ultimate Tensile Strength (N)	Elongation at Break (%)
H-AGMA1	30 ± 0	6 ± 0	0.55 ± 0.06	0.3 ± 0.1	0.04 ± 0.01	0.10 ± 0.06	5.3 ± 3.1
Silk	40.6 ± 1.9	6.7 ± 1.0	0.21 ± 0	192.7 ± 55.4	1.96 ± 0.60	2.79 ± 0.43	6.8 ± 2.1
Silk/H-AGMA1	34 ± 0.12	7.2 ± 0.5	0.13 ± 0.01	162.7 ± 17.8	2.58 ± 0.87	1.95 ± 0.62	8.2 ± 1.9

## Data Availability

Not applicable.
